# Comprehensive survey of United States internet users’ sentiments towards cryopreservation

**DOI:** 10.1371/journal.pone.0244980

**Published:** 2021-01-07

**Authors:** Christopher Robert Gillett, Taylor Brame, Emil F. Kendiorra

**Affiliations:** European Biostasis Foundation, Riehen, Canton of Basel-Stadt, Switzerland; University of Bologna, ITALY

## Abstract

Medical cryopreservation is the speculative practice of using low temperatures and medical-grade cryoprotective agents to halt the decay of a recently-deceased person’s brain and body for the prospect of future resuscitation and restoration of function. We conducted a survey of 1,487 internet users in the United States to measure familiarity with, interest in, beliefs about, and attitudes towards cryopreservation. The majority of respondents (75%) had previously heard of the topic. Respondents tended to underestimate the cost of cryopreservation and number of previous cases but overestimate the number of providers. While many respondents expressed interest in signing up (20%) or had actively researched the topic (21%), a much smaller fraction have decided to be cryopreserved (6%). This level of interest is much greater than the number of previous preservation cases would indicate. We found that respondents’ attitudes towards death significantly correlated with their general sentiments towards the topic, with those expressing a desire for longer life or to see the future being more interested and positively inclined. Fear of death was not associated with interest in cryopreservation. Negative sentiments towards cryopreservation were less common than respondents perceived. For example, 14% of respondents believed that “most people” think cryopreservation should be illegal, but only 4% of respondents actually did. Many respondents (42%) were pessimistic regarding the likelihood of cryopreservation being successful, but the mean estimate of time until revival of cryopreserved bodies would be possible was 82 years.

## Introduction

Inducing metabolic stasis (“biostasis”) in humans using cryogenic temperatures was proposed in the 1960s as a way of preventing structural decay of the brain following the terminal failure of the body’s critical systems [1, 2]. Indefinitely preserving the brain’s physical state leaves open the possibility of “resuscitating consciousness” by restoring function to the brain using one of several theorized but as of yet undeveloped techniques such as digital reconstruction or nanobot cellular repair [3]. Because bodies can remain in biostasis indefinitely, patients preserved prior to the development of these theorized techniques would still benefit, thus there is a rationale for conducting cryopreservation on a speculative basis [1, 2].

The first cryopreservation operation was performed in 1967, and that body remains in biostasis under the care of one of the two major cryopreservation providers in the United States [4]. The marginal community of cryopreservation institutions, researchers, customers, and activists have had an eventful history in the intervening decades, periodically appearing in the press because of various controversies and legal challenges [5].

The concept has become a popular plot device in fiction, but the dramatic depictions often differ from the realities of cryopreservation as it is currently practiced. Cryopreservation is most often depicted as an easily reversible indefinite hibernation, made possible by a “cryo-chamber”, such as in *2001*: *A Space Odyssey* (1968) or *Futurama* (1999).

Despite widespread exposure to cryopreservation in fiction and news media, exceedingly few people have ever arranged the procedure for themselves. Approximately 400 preservations have been done, some craniums and some whole bodies, and about 3,100 living people maintain legal and financial arrangements to make cryopreservation a part of their end-of-life care [6–8]. Saul Kent, a prominent funder of cryopreservation research, has stated his belief that the topic “has received more publicity with less results than any idea in history” [9].

Cryopreservation has had a long-standing presence in culture, and challenges popular conceptions of the very nature of death, but very little is known empirically about the general public’s awareness of, attitudes towards, beliefs about, and personal interest in the topic.

As consumer demand shifts from burial to cremation [10], a new, more comprehensive survey of the public’s associations with cryopreservation is needed, both to aid in messaging the practice and to contribute to sociological research concerning medicine and death.

## Methodology

The European Biostasis Foundation’s Institutional Review Board, a group of internal members independent of the authors, approved the study protocol. Participants were informed that participation was completely voluntary and they could withdraw from participation at any time. All data was processed anonymously, and all participants were adults.

### Data collection

Participants were recruited with posts on the community classified ads website Craigslist over the first two weeks of July, 2020. Advertisements offering the chance to win a $100 Amazon gift card in exchange for completing a survey were posted to 36 of the largest metropolitan areas in the United States. The online questionnaire builder Paperform [11] was used to collect responses. Participation was voluntary, all respondents were 18 years of age of older and consented to participate, and all data was processed anonymously. Participants self-reported their age, understanding that this would be verified if they were selected to receive a financial prize. In total 1,571 responses were collected, and 1,478 remained after invalid responses were removed.

### Survey and question design

Upon opening the survey webpage, participants were first provided the following description of cryopreservation:

*Please share your perspective on an experimental practice of emergency medicine known as Biostasis / Cryopreservation or "Cryonics"*.

*Biostasis / Cryopreservation is a method of ‘freezing’ people who have just died with the aim of ‘reviving’ these persons in the future*—*with the help of more advanced medicine*.

*In an ideal case*, *a cryopreservation team begins the procedure at the moment the patient’s heart stops*. *They quickly restart circulation as they lower the body’s temperature to just above freezing*, *apply medical-grade anti-freeze*, *then bring the body’s temperature to -196 degrees Celsius for long-term storage in liquid nitrogen*.

*The oldest cryopreservation provider is a non*-*profit that has been operating continuously for 48 years*.

*Hypothetically*, *a well-preserved brain may have enough information for future scientists to read memories or restore consciousness*.

Questions were organized into 1) demographics and background, 2) death attitudes and end-of-life plans, 3) cryopreservation knowledge quiz, and 4) cryopreservation sentiment. Response formats included numerical, choosing one response from a list (including yes/no and agree/disagree on a 1–5 Likert scale), and allowing multiple selections. All questions and corresponding answer options are included as S1 File.

### Statistical analysis

The results were exported from Paperform into CSV format. Using pandas, a popular open source data analysis library for the programming language Python [12], obviously invalid responses were removed. Some numeric variables were bucketed into categorical variables to make interpretation of results clearer. Likert scale responses (agree/disagree on a 1–5 scale) were converted to numbers from 1 to 5, with 5 indicating strong agreement. The variable “economic achievement” was created by assigning each of the six income categories ($20,000 to $100,000 in $15,000 increments) a number and dividing each respondent’s income bracket by their age, thus controlling for the effect of age on income.

Scipy, a statistical functions library for Python [13], was used to generate a Pearson correlation coefficient and corresponding P value, two-tailed, for variables of interest. These P values were then adjusted for multiple testing with the Bonferroni method, using the statsmodels Python library [14]. Percentage values are rounded to the nearest full number. P values below 0.01 were considered to be significant, and P values shown are rounded to the .0000 decimal place.

## Results

### Demographic effects

Of the 1,478 responses, women accounted for 51% of responses, men for 47%, and 2% of respondents gave an alternative response—roughly in line with the gender ratio of the United States [15]. Ages ranged from 18 to 80, the median being 33 years old, and the median age was the same for men and women.

Because of how responses were solicited, the dataset includes a sample bias. Respondents are younger, experiencing greater economic insecurity, and more comfortable with technology than the general American public. Eliminating those under the age of 18, the 2010 US Census found 54% of Americans were between the ages of 18 and 44 [15], but 77% of respondents were in that range. The US Census Bureau found in 2018 that 15% of Americans earn less than $20,000 [16], much fewer than the 32% of respondents who reported being in that income bracket. 96% of respondents agreed that they are “tech-savvy”. This high tech literacy represents a relevant divergence from a representative sample of the US population [17], with only 20% correctly answering seven or more questions designed to test general digital proficiency in a Pew study. Therefore, the sample should be considered “US internet users”.

It is also necessary to consider more subtle biases that may exist. For example, although the title of the survey solicitation mentioned only a generic survey and the possibility of payment, the body of the advertisement said that it was concerning “brain freezing”. People who are interested in the concept would be more interested in participating. However, this doesn’t imply that our responses would be more positive, only that respondents may be more likely to have pre-existing exposure to and opinions about the topic. Furthermore, responses were incentivised with the possibility of winning a monetary reward. This would attract people who are more inclined to wager, and such people may have a “gambler’s mindset” which would make them more open to the speculative nature of the “cryopreservation gamble”. The advertisement was not targeted based on any algorithm, and all website users were equally likely to view the post at any given time.

Regarding demographic effects, men’s responses diverged from women’s. As shown in Table 1, men reported significantly more familiarity with the topic, more interest, and more optimism that the procedure will be successful. Women were more in favor of making the practice illegal, although only 4% of women selected that option in total. With regards to death attitudes, men expressed more desire to extend their lives beyond what is possible—34% desire to live indefinitely.

**Table 1:** Demographic factors and general sentiment correlations.

	Men	Women	Low economic achievement	High economic achievement	<30 years old	>64 years old
**Heard more than most**	.102**	-.108**	.004	.04	.032	.023
**Not signed up, but interested**	.125**	-.112**	-.076*	.087**	-.019	-.044
**Good chance it works**	.109**	-.093**	.037	-.006	-.063	.038
**Should be illegal**	-.15**	.151**	.058	-.065	-.009	.018
**Wish to live forever**	.156**	-.157**	-.051	.078**	-.022	-.012

Pearson correlations between demographics and general sentiment.

* = 0.05 significance and

** = 0.01 significance.

Those with low economic achievement (having lower incomes, controlled for age) expressed less interest in seeing “what the future will be like” (R^2^ = -.115, P = .0002), and those with higher achievement expressed greater interest—37% versus 53%. Despite their greater interest, high economic achievers also expressed the concern, 43% in total, that “the chance that it will work is low” (R^2^ = .117, P = .0003). The middle third of respondents by economic achievement did not differ significantly.

While respondents don’t differ significantly in their general sentiments by age, they do diverge in other ways. Respondents under the age of 30 are more likely to have not made end-of-life decisions and have no plans to do so soon (R^2^ = .179, P = .0000). They more frequently stated the belief that “most people” think cryopreservation is “cool and exciting, if quite unusual” (R^2^ = .182, P = .0000) but also “dystopian” (R^2^ = .257, P = .000). As expected, those 65 years or older were more likely to have formalized their end-of-life wishes in the form of a will (R^2^ = .146, P = .0004).

Table 1 shows how three demographic factors, gender, income, and age, correlate with a range of other survey answers. Based on their answers to demographic questions, respondents were sorted into groups, represented by columns in the table. The rows are other survey answers. The highlighted cells indicate a statistically significant correlation between the demographic group of the column and the survey answer of the row. Economic achievement is the ratio of a respondents income to age, and respondents were sorted into three buckets based on their percentile of economic achievement. The table shows two age groups: those under the age of 30 (36%), and those over the age of 64 (2%). These groups were chosen because they correspond to distinct life stages. Other age groups are not shown, but they likewise have no statistically significant relationship with the listed questions.

### Attitudes towards death

A respondent’s attitude towards death was found to significantly correspond to their interest in cryopreservation. Table 2 shows the correlation between death attitudes and three questions indicating general sentiments: whether one believes the practice should be banned, whether one agreed with a statement of mild interest, and whether one has researched the topic before.

**Table 2:** Death attitudes and general sentiment correlations.

	Believe in afterlife	Death frightens me	I wish to see the future	Pro-longevity	Anti-longevity
**Should be illegal**	0.081**	-0.017	-0.163**	-0.208**	0.167**
**Exciting and I will research**	-0.086**	0.031	0.164**	0.217**	-0.239**
**Have researched before**	-0.046	0.000	0.094**	0.03	-0.103**

Pearson correlations between death attitudes and general sentiments.

* = 0.05 significance and

** = 0.01 significance.

Respondents were asked with a Likert scale question if they believe in an afterlife. Condensing all responses indicating agreement, and all responses indicating disagreement, 43% agreed/strongly agreed, 33% were unsure, and 24% disagreed/strongly disagreed. Those who believed in an afterlife were less likely to have heard of cryopreservation (R^2^ = .118, P = .0001). Contrary to the prior assumption of the authors, not believing in an afterlife was not significantly correlated with a desire for cryopreservation.

18% selected that “death frightens” them. 45% selected that they wish to “see what the future will be like”. Two statements represented “pro-longevity” sentiments, “if I could remain youthful for another 100 years I would” and “If I could live indefinitely, even ‘forever’, I would”—selected by 44% and 26% of respondents, respectively. Another two statements represented “anti-longevity” sentiments, “death will always be inevitable and science will never be able to change that” and “death is natural and should not be avoided”—selected by 10% and 29%, respectively.

People who selected more “pro-longevity” attitudes had significantly more positive sentiments. They tended to agree “I would be interested in talking to a cryopreservation company about signing up” (R^2^ = .211, P = .0000)—of those who had at least one pro-longevity attitude, 46% agreed with the statement. They also tended to say that “there’s a good chance cryopreservation will work” (R^2^ = .208, P = .0000) and that they wish to “see what the future will be like” (R^2^ = .285, P = .0000).

People who had more “anti-longevity” attitudes tended to say that cryopreservation was “unlikely to work” (R^2^ = .231, P = .0000), that cryopreservation “makes death harder” for loved ones (R^2^ = .212, P = .0000), and that they’re “not interested” in signing up (R^2^ = .206, P = .0000).

People who reported fear of death expressed more concerns about cryopreservation, in particular that the procedure will “hurt or be traumatic” (R^2^ = .146, P = .0000) and their loved ones would not be with them when their consciousness is resuscitated from biostasis (R^2^ = .162, P = .0000). The hypothesis that this group would be more likely to agree with the statement “cryopreservation is an exciting idea and I intend on looking into it further” was rejected (R^2^ = .031, P = .6383).

Those who wish to see the future tended to agree that “cryopreservation is exciting” (R^2^ = .181, P = .0000) and to say that “financial stability” is important to them in selecting a cryopreservation provider (R^2^ = .206, P = .0000).

### General exposure and sentiment

The vast majority of respondents reported some familiarity with cryopreservation—64% said they’d heard “about as much as most people” while 11% said they’d heard more than that. Only 20% had not heard about cryopreservation at all.

The most common media sources that respondents reported learning about cryopreservation from were fictional films and internet articles, with 50% and 48% selecting those options, respectively. Of all respondents, only 6% reported getting information from a cryopreservation provider’s website, and 21% had actively sought out information about cryopreservation. Those who had researched the topic were more likely to have learned from a cryopreservation provider website (R^2^ = .315, P = .0000).

When asked if they were signed up for cryopreservation, 20% of respondents said “no, but I’m interested”, 9% said “no, and I’m not interested”, and 47% gave the noncommittal response “no, I’ve never thought about it before”. The remainder wrote in their own answer or responded “none of the above” or “prefer not to answer”. To the statement “I believe that cryopreservation is an exciting idea and intend on looking into it further”, responses were collected on a 1–5 Likert scale (agree/disagree). 48% agreed/strongly agreed and 15% disagreed/strongly disagreed. There was weaker agreement with the stronger statement, “I would be interested in talking to a cryopreservation company to discuss signing up”—39% agreed and 24% disagreed. It seems that the public is largely open to the topic but has not thought seriously about it as a realistic alternative to burial or cremation.

In order to determine each respondent’s stage of purchase intention regarding end-of-life wishes, they were asked whether they had decided what they wanted to happen to their body when they die (burial, cremation, or cryopreservation), whether they planned to make a decision soon if not, and whether they’d formalized their decision (told family, made a will, etc). Surprisingly, 6% of respondents indicated that they intend to be cryopreserved upon their death—though 81% of them have taken no steps to formalize this intention. 3% reported being a member of a “longevity or life extension” community.

One way to estimate the size of the receptive population is to eliminate those who have decided on or formalized end-of-life wishes other than cryopreservation, as well as those who indicated “no interest” in cryopreservation. This suggests that up to 28% of respondents might be interested and choose to sign up in the future.

Next, to evaluate respondents’ factual knowledge, questions about cryopreservation as it is practiced today were asked. Respondents tended to overestimate the number of providers. 25% estimated that there are between 2 and 3 providers, the roughly correct range [18]. 16% underestimated, and 59% overestimated.

A similar portion, 26%, correctly identified the purpose of “vitrification” to be “to reduce ice formation” [19]. The most popular response, selected by 32%, was “I don’t know”.

Asked about the number of cryopreserved bodied, only 7% guessed within the roughly correct range of 300 to 600 [6, 8]. 57% underestimated, and 36% over estimated.

A high proportion also underestimated the price of cryopreservation today. Depending on the provider, the service package selected, and the method of funding the price one pays typically ranges from $25k to $300k [20, 21]. 40% guessed below $25k. 17% guessed within the correct lower range of $25k-$85k. 25% guessed within the correct higher range of $85k-$300k. The remaining 17% overestimated.

Although respondents underestimate the price of cryopreservation, the perception that the procedure is excessively expensive was reported by many respondents. This perception was selected by 47% as one of the main “problems with cryopreservation today”, and 39% said one reason they had not signed up was because of excessive cost. 37% said they may sign up if the cost were the same as cremation.

Out of four questions, 23% got none correct, 42% got one correct, 27% get two correct, 7% got three correct, and 1% got all four correct.

As expected, higher quiz scores were associated with respondents who reported having actively researched cryopreservation in the past (R^2^ = .104, P = .0046).

Several questions measured respondents’ preferences regarding end-of-life care. Cost, family’s wishes, convenience, and quality were the top four most selected considerations regarding end-of-life care, selected by 58%, 40%, 33%, and 32%, respectively.

When asked why they hadn’t yet signed up, the most common answer was that the respondent had “never thought about it until now”—49%. Also common was that it is “too expensive” (39%), “too complex or I don’t know how” to sign up (23%), and that the respondent could “do it later” (18%). Social stigma and mistrust of providers were minor concerns, selected by only 5% and 7%, respectively.

The plurality of respondents, 45%, identified “it is too expensive” as one of the biggest problems with cryopreservation. Other option choices included “the company might go out of business” (35%), “social stigma” (31%), “few scientists and doctors recommend” (29%), “damages the body too much” (25%), and “no one in the future will want to revive the bodies” (18%).

Respondents greatest concerns about cryopreservation were that loved ones would not be with them when they are resuscitated (45%), “the chance that it will work is low” (32%), they would not understand the society they wake up in (29%), and that it will “hurt or be traumatic” (28%).

People indicated that they would be “significantly more likely to sign up” if they could specify under what conditions they are to be revived (44%), the cost were the same as cremation (37%), and their loved ones signed up too (29%).

In the minds of the respondents, the “most significant risks” to revival were that the company “goes out of business” (48%), “revival is too expensive” (47%), “future society does not develop technologically” (43%), and “cryopreservation is made illegal” (33%). Scientific risks, such as that “current cryopreservation is too crude” (25%) and “it is impossible in principle” (17%), were selected the least.

The most important considerations when choosing a cryopreservation provider were “cost” (60%), “research” (59%), “track record” such as the age of the provider organization and the number of preservations it had performed (48%), and “financial stability” (44%).

### Concerns and objections

Some attention was devoted to ethical issues related to the practice of cryopreservation. Questions focused on moral considerations that would be relevant to families and patients. In general, broader social implications, like those explored by Dr. Francesca Minerva in her book *The Ethics of Cryonics*, were not included, although participants were asked their opinion on the possibility of longevity technology contributing to overpopulation. The concerns regarding medical biostasis highlighted in the survey include some but not all of the common ethics arguments identified by Shaw (2009) [22]. Participants were specifically asked about the effect the practice has on surviving loved ones and the perception that it is against nature. Shaw’s more comprehensive analysis included environmental, financial resource usage tradeoff, and organ donation considerations. Because of practical limitations on the length of the survey, these issues were not specifically referenced and instead participants’ approval or disapproval on moral grounds was gauged with more general questions.

Respondents were asked on the Likert scale whether they agreed or disagreed with the statement “cryopreservation should be illegal”. The vast majority disagreed (36% “disagree” and 23% “strongly disagree”), and 36% were unsure. Only 4% agreed with the statement whatsoever. Those who supported making the practice illegal were significantly less likely to have actively researched it before (R^2^ = .169, P = .0000). Only 17% of those who supported banning the practice had researched the topic previously. Of the entire dataset, slightly less than 1% had both researched cryopreservation and agreed that it should be illegal. When all participants were asked whether they think most other people would support making cryopreservation illegal, 14% agreed. This is a noteworthy finding because it indicates that public sentiment towards cryopreservation is significantly more positive than is generally believed. Furthermore, 32% agreed that “most people” think cryopreservation is a “scam/rip-off”, but when asked why they haven’t signed up yet, only 7% said it’s because they didn’t trust the providers. Two question options gave participants the option to express distrust of cryopreservation providers, of which 8% of respondents selected at least one.

Few people, 3%, agreed with the statement that “cryopreservation is immoral”. Those who did were more likely to agree that the practice should be illegal (R^2^ = .157, P = .0000). A common objection to longevity is the potential for overpopulation [23], but only 10% agreed that “cryopreservation will create overpopulation”. To the statement “Cryopreservation makes death harder for my loved ones”, 9% agreed. People who expressed this concern tended strongly to select “social stigma” as a reason against having not signed up yet (R^2^ = .252, P = .0002).

As shown in Table 3, all of these negative sentiments were negatively correlated with interest in talking to a provider about signing up, positively correlated with the belief that the practice is unlikely to work, and positively correlated with the attitude that death is natural and shouldn’t be avoided. Also noteworthy is that all these negative sentiments are positively correlated with each other, significant at the 0.01 level (except the correlation between “you can’t trust most cryopreservation companies” and “cryopreservation should be illegal”).

**Table 3:** Negative sentiments and other attitudes correlations.

	You can’t trust most providers	It is immoral	Will create over population	Hurts loved ones	Should be illegal
**Interested in talking to a provider**	-.093**	-.133**	-.113**	-.157**	-.288**
**Unlikely to work**	.247**	.102**	.113**	.219**	.126**
**Death is natural, shouldn’t avoid**	.107**	.122**	.127**	.183**	.166**

Pearson correlations between negative sentiments and other attitudes.

* = 0.05 significance and

** = 0.01 significance.

Next, respondents were asked to choose statements about cryopreservation that they believed most people—not necessary themselves—would agree with. 32% selected that most people consider it a “scam/rip-off”, 45% selected “weird”, 30% selected “dystopian”, and 12% selected “selfish”. All these sentiments, except for “dystopian” were significantly positively correlated with each other, except that “dystopian” is not correlated with “should be illegal”.

The perception that the practice is widely seen as “dystopian” was heavily associated with respondents under the age of 30 (R^2^ = .257, P = .0000). Those who selected “scam/rip-off”, for example, tended to be concerned that “the chance that it will work is low” (R^2^ = .254, P = .0000), “current cryopreservation is too crude” (R^2^ = .208, P = .0000), the “company might go out of business” (R^2^ = .201, P = .0000).

### Perception of likelihood of success

Three questions gave respondents an opportunity to express pessimism about the prospects of the cryopreservation endeavor. 41% selected at least one of the three pessimistic statements.

Respondents who selected more of the three pessimistic answers tended to hold the opinion that a significant risk to the endeavor was that “current cryopreservation is too crude” (R^2^ = .292, P = .0000).

The more optimistic statement “there’s a good chance cryopreservation will work” was selected by 44% of respondents. Those who agreed were significantly more likely to indicate that they are interested in cryopreservation (R^2^ = .250, P = .0000), to agree with the statement that “cryopreservation is an exciting idea and I intend on looking into it further” (R^2^ = .342, P = .0000), to agree with the statement “I would be interested in talking to a cryopreservation company to discuss signing up” (R^2^ = .321, P = .0000), and to express the desire to “live indefinitely” (R^2^ = .208, P = .0000). They also tended to disagree that cryopreservation should be illegal (R^2^ = .226, P = .0000).

The question “in how many years do you think it will be possible to revive a cryopreserved body, in an ideal case?” accepted any numerical answer. After removing responses greater than 2 sigma, which were determined to be invalid, the mean response was 82 years and the median was 40 years. 43% estimated 25 years or less, 21% estimated 26 to 50 years, 19% estimated 51 to 100 years, and 8% estimated 100 to 200 years, and 8% estimated greater than 200 years (including the responses that were eliminated for computing the mean).

## Discussion

To our knowledge, this study with 1,478 responses represents the largest data set across the United States concerning cryopreservation.

### Considerations on methodology

It was determined that an introductory explanation was needed to ensure that respondents share a basic familiarity with the topic so that results would not be needlessly diluted by respondents having no or a very limited prior exposure to the topic, increasing the chance that they select options at random. The description was crafted to provide exposure to the theoretical, operational, and institutional aspects of cryopreservation, making clear that while the procedure is speculative it is practiced with a degree of professionalism. The initial definition is similar to the one used by Kaiser, et al. [24], to allow for some comparability. Many questions about cryopreservation also serve to make respondents aware of the range of considerations to weigh when deciding whether the operation is a good fit for them before asking about their overall interest in signing up at the end of the survey.

As discussed in the results section, there was a bias towards people who are younger, lower-income, and more comfortable with the internet. We determined that attempting to adjust for these biases would be too imperfect to be warranted and thus we present our results with the caveat that the sample is biased in the ways described.

### Interpretation of results

We confirmed the existing perception [25] that men have more positive sentiments towards cryopreservation than women. A meta-analysis of an online science and philosophy community survey known to be enthusiastic about the topic, Less Wrong, has previously found the same [26]. It is noteworthy that one of the most well known cases of cryopreservation was a young woman [27], and a woman co-founded the longest-lasting cryopreservation institution [28].

Our observation of the relationship between income (adjusted for age) somewhat challenges the popular notion that cryopreservation is “for the wealthy” [29]. Although there was a significant correlation between high economic achievement and interest in the topic, a finding counter to that of the Less Wrong meta-analysis [25], the correlation between high income and desire to see the future was much stronger. It may be that the tendency of the wealthier to express interest in cryopreservation is a consequence of their more “pro-longevity” attitudes, perhaps because of greater life satisfaction and a different hierarchy of needs. Wealthier respondents weren’t more optimistic about the likelihood of success, but perhaps because of their greater means and life satisfaction, they tend to view cryopreservation more favorably from a risk versus reward perspective.

This survey lends support to Kaiser et al.’s finding that the average income of those interested in cryopreservation was €20,000 to €30,000. The plurality of our respondents saying they’re “interested” in signing up fell in the $20,000 to $34,999 income bracket.

Despite evidence that one’s fear of death tends to differ with age [30], no significant differences between general sentiments and age were observed. Younger respondents weren’t more interested in cryopreservation, counter to the results of the Less Wrong meta-analysis [26]. However, certain significant age effects were present. Respondents diverged in their end-of-life planning by age, likely because of the importance of one’s stage of life in making end-of-life plans. Most interesting was the perception of younger people that “most people” believe that cryopreservation is “cool and exciting, if quite unusual” but also “dystopian”. It makes sense that young people, being more steeped in a culture that is tech-savvy, interested in “the next big thing”, and open to questioning tradition, would perceive that the popular attitude towards cryopreservation is that it is “cool and exciting”, but “dystopian” is less obvious. The age difference in the perception of cryopreservation as “dystopian” may indicate that the science fiction that young people watch is more pessimistic than older science fiction. One of the most popular science fiction programs in recent years is *Black Mirror* (2011), which introduces futurist concepts, with a dark twist.

Attitudes towards death and conceptions of the afterlife were strong indicators of a variety of variables related to cryopreservation. Those who believed in an afterlife were less likely to have heard of the practice before, perhaps because, if cryopreservation and a religious afterlife are competing notions of how one should deal with their mortality, those who have chosen religion have no need to “comparison shop”.

We found similar rates of various death attitudes to Kaiser et al.’s findings. Kaiser et al. found that 13% reported above average fear of death. This study saw 18% of respondents indicate that death frightens them. Kaiser et al. posed the statement “death is a problem that needs to be solved”, which 31% of respondents agreed with. That exact statement was not included in this study, but the two questions measuring interest in medical alternatives to aging and death—“if I could remain youthful for another 100 years I would” and “If I could live indefinitely, even ‘forever’, I would”—receive 44% and 26% agreement, respectively. This is lower than the 37% that Kaiser et al. found wished to “live indefinitely”.

Pro-longevity attitudes were significantly correlated with interest in biostasis in both this survey and Kaiser’s. For example, Kaiser et al. finds that those who said “I want to (physically) live forever” were more likely, significant at the 0.01 level, to find cryopreservation “conceivable” for themselves (R^2^ = .207). This study likewise found that those who express similar pro-longevity sentiments are significantly more likely to report being interested in talking to a cryopreservation company about signing up (R^2^ = .211, P = .000).

We observed no significant correlation between fear of death and any of the many variables indicating interest in or positive sentiments towards cryopreservation, strongly disconfirming the popular conception that people are interested in cryopreservation because they fear death [31]. If media coverage of cryopreservation gives this impression, it may be that the most visible members of the community tend to be motivated by death anxiety, but the average cryopreservation subject is not. In fact, people reporting greater fear of death tended to express more concerns about the practice, indicating that their anxieties make it difficult for them to rationalize the uncertainties inherent in cryopreservation.

We found much more awareness of the topic than Kaiser, et al. found—75% compared to their finding of 47%, likely because most of the history of cryopreservation has taken place in the United States and the news coverage that activity generates has increased awareness in the country [32].

As expected, the public’s most common sources of information about cryopreservation were fictional films and internet articles. We can infer that most of the public’s exposure comes from a handful of movies and TV shows, most of which depict the process as similar to hibernation in a “cryo-chamber”. These media typically do not depict the uncertainties inherent in the enterprise, so many viewers will be unfamiliar with the rationale behind performing preservation while resuscitation techniques are still only theoretical. Internet articles typically cover specific news stories, such as legal disputes and controversies, or general introductions to the topic.

Many questions served in part to indicate the respondent’s level of interest in cryopreservation. In general, 40%-50% of people registered some level of interest, 20%-30% registered moderate interest, and about 5% registered strong interest.

The weakest statement of interest—“cryopreservation is an exciting idea and I intend on looking into it further”—was agreed to by 48%. 39% said they would be interested in talking to a cryopreservation provider. The ceiling of interest is likely in this range, with the remaining respondents having death attitudes that are incompatible with the practice. For example, 29% agreed that “death is natural and should not be avoided”. These people were significantly less likely to express interest in cryopreservation. Additionally, 43% expressed belief in an afterlife.

A smaller but still substantial group showed stronger interest. 21% had actively researched the topic previously. 25% said they were “not signed up, but interested”. Kaiser et al. measured general interest slightly differently, with the question “could you imagine having your body ‘cryonized’ immediately after your death?”, but got a similar result as 24% said yes. The proportion of the population that might develop an intention to be cryopreserved is likely in this range.

The strongest indications of interest were having visited a cryopreservation provider’s website—6% had done so—and personally intending to be cryopreserved upon death—6% said this. 3% reported being a member of a “longevity or life extension” community. Another survey in 2019 found that 3% of respondents intended to opt for “body preservation (i.e. cryogenesis)” instead of a more traditional burial method [33]. This is half what this study found—6%—but these results together begin to point at a likely range for the size of organic high-intention interest in cryopreservation.

If 6% of Americans really intend to be cryopreserved, nearly 21 million people in the United States, or 18.9 million if only counting internet users (which was our sample), currently intend to opt for cryopreservation when they die. Given this large population of informed, high-intention consumers, mere exposure is not the limiting factor in the adoption of cryopreservation. If 21 million Americans really intend to be cryopreserved, and only 3,100 have actually made such arrangements, only 1 in 6,774 people who might intend to be cryopreserved have made formal arrangements. This sign-up rate is so low that even if the remaining 20% of people who have not heard about the topic were exposed to the idea, it would not do much to make cryopreservation a mainstream end-of-life choice on the scale of burial or cremation.

The cryopreservation community has long been frustrated and confused by how few people actually make arrangements [9], given how many people express at least some interest and that they “have nothing to lose”. With new empirical findings confirming a dramatic discrepancy between the volume self-reported interest and the actual number of cryopreservation operations, this question deserves consideration.

One way to approach the question is to get a better understanding of the public’s preconceptions of cryopreservation. Our knowledge test showed that the public generally underestimates the cost of cryopreservation and the number of cryopreserved bodies, but overestimates the number of providers. The view of the plurality may be that the cryopreservation ecosystem is composed of a large number of low-cost providers that have performed few preservations. This indicates that the routine, standardized, and institutionalized service that cryopreservation providers aspire to (but have not achieved to date) has indeed generally not entered the public’s awareness.

Participants identified cost as a major factor in their decision-making regarding cryopreservation in every question where cost was an answer choice. The plurality of respondents, 60%, said that cost was one of the most important considerations in choosing a cryopreservation provider. A plurality, 58%, also selected cost as the main consideration in end-of-life plans generally. When asked what, if anything, would make them “significantly more likely to sign up”, 37% chose if “the cost were the same as cremation”, making it the second most selected choice. Depending on the provider, service package, and funding method one chooses, the cost of cryopreservation can vary from around $20,000, $80,000, or $200,000, whereas cremation typically costs around $4,500.

Badger [34] presented participants with several hypotheticals, asking them which would make them most favorable to cryopreservation. In order of persuasiveness, these included “if it were cheaper”, “someone in my family were signing up”, “my physician approved”, and “millions of other people were signing up”. We presented respondents with hypotheticals that tested the impact of similar underlying motivations, and we found that their persuasiveness ranks in the following order, starting with most persuasive: “it costs the same as cremation”, “my loved ones sign up too”, “a major healthcare provider offered” it, and “my physician recommended it”. This study generally confirms the hierarchy of motivation found in Badger’s survey. Indicating that cost and social acceptance being the most important factors.

As for more negative perceptions of cryopreservation, the community as a whole is perennially concerned that public attention could result in the practice being banned or otherwise negatively impacted, a fear that causes some to reject the vision of large-scale, highly institutionalized cryopreservation operations. In his paper “The growth and decline of cryonics”, David Sanders Stodolsky says there is “little doubt that the current status of cryonics is that of a cult in the eyes of the general public” [35]. This survey indicates that fear of such perceptions is not warranted.

Only 4% of respondents agreed whatsoever that cryopreservation should be illegal. Those who held this opinion were significantly more likely to have never researched the topic before—of this group only 17% had. Because they are not informed about the topic, we may infer that they do not hold the opinion strongly and are not motivated to support an actual effort to restrict the practice of cryopreservation. Those who agreed that the practice should be illegal were significantly more likely to have “anti-longevity” death attitudes and to agree that the practice is “immoral”, but they don’t tend to select specific objections such as that the practice makes death harder for loved ones or would lead to overpopulation. This likewise indicates that the group has not thought much about the topic but rather has a vague, intuitive disinclination towards it because of their personal attitudes towards death. Much of the same is true for the 36% of people who are “not sure” if the practice should be illegal.

With regards to potential social harms, participants were asked if they agree that it could lead to overpopulation and if they think it would make death harder for loved ones of the deceased. Few agreed—10% and 9%, respectively. Similar to the questions about legality, there does not seem to be relevant animosities in public opinion.

The fear of overpopulation has been identified as one of the major objections to longevity in general [23]. Longevity advocates generally do not share this concern because they tend to hold optimistic views about future material conditions, thus they feel that the future will be prosperous despite unprecedented population growth [36]. This survey likewise finds that members of the public who “wish to see the future”—most of whom can be assumed to expect to see a positive, prosperous future—are significantly more likely to express positive sentiments towards cryopreservation. Popular conceptions of what the future will be like heavily influence public interest in cryopreservation.

If one thinks that medical cryopreservation has a significant possibility of working, these incidental drawbacks are not important, but if one sees the likelihood of success as almost 0%, the practice may be hard to justify given the harms. As expected, people who were concerned about making death harder for loved ones were significantly more likely to be pessimistic about the success of cryopreservation. They were also significantly more likely to express concern about social stigma and to express mistrust of providers.

Only 8% of people selected one of the two answer options to indicate mistrust of cryopreservation providers, but 32% of respondents believed that “most people feel” that cryopreservation is a “scam/rip-off”. This is more than “immoral”, selected by 12%, (though less than “weird”, selected by 45%) indicating that this is more significant than the previously discussed perceptions of broader social harm.

Those who agreed that “most people feel that cryopreservation is a scam/rip-off” were significantly more likely to be pessimistic about the chance of success, to say that the practice is currently “too crude”, to identify organizational stability as a risk, to doubt that people in the future will want to revive the bodies, and to agree “you can’t trust most cryopreservation companies”. This study indicates that, rather than seeing cryopreservation providers as fraudsters, a typical attitude towards providers is that they are well-intentioned but selling an low probability hope at too high a price.

It’s noteworthy that although many respondents said that “most people” believe that the practice is a “scam/ripoff”, very few had negative sentiments themselves, indicating a relevant misconception about what peers think about the practice. The general public overestimates the degree of negative sentiment towards the practice. Referring back to the results of the knowledge questions, this may be because the general impression of the ecosystem is that it consists of many low-cost, inexperienced operators. Such a landscape would certainly be more likely to include dubious operators than the more centralized, institutionalized market that exists today. The earlier days of cryopreservation more resemble the former than the letter, when many small groups were interested in performing “garage science project” cryopreservations. The public’s impressions of cryopreservation may be greatly out of date.

In this study we present an in-depth sentiment analysis, confirming results from previous publications in a larger data set while providing a more comprehensive understanding of the motivation that leads people to be interested in cryopreservation. In future studies, we plan to elucidate the difference between the surprisingly high interest in the topic and the comparatively very low rate of sign ups.

## Supporting information

S1 File Survey questions and answer choice. (DOCX)

S2 File Survey results. (XLSX)
