# Correction: Comprehensive survey of United States internet users’ sentiments towards cryopreservation

**DOI:** 10.1371/journal.pone.0251225

**Published:** 2021-04-29

**Authors:** Christopher Robert Gillett, Taylor Brame, Emil F. Kendziorra

The third author’s name is spelled incorrectly. The correct name is: Emil F. Kendziorra. The correct citation is: Gillett CR, Brame T, Kendziorra EF (2021) Comprehensive survey of United States internet users’ sentiments towards cryopreservation. PLoS ONE 16(1): e0244980. https://doi.org/10.1371/journal.pone.0244980
