# Supplementary material for: Comprehensive survey of United States internet users’ sentiments towards cryopreservation
Source: PLoS One. 2021 Jan 7;16(1):e0244980. doi: 10.1371/journal.pone.0244980 (PMC7790260; doi:10.1371/journal.pone.0244980)
Supplement: S1 File — (DOCX) [file pone.0244980.s001.docx]

| **Age**  Numerical response |
| --- |
| **Gender**  Select one  Male  Female  Other (specify) |
| **Occupation**  Select one  Retired  Seeking work  Homemaker  Management  Business/Finance  Computers/math  Architecture/engineering  Life, physical, or social science  Community and social services  Law  Education  Arts, design, entertainment  Healthcare  Police/fire/EMS  Food preparation and service  Building maintenance  Personal care services  Sales and related  Office and admin. support  Farming, fishing, forestry  Construction and extraction  Installation, maintenance, repair  Production and manufacturing  Transportation and moving  Military  Student  Other (specify) |
| **Country of residence**  Select one  (lots of options lol) |
| **Education**  Select one  Less than high school  High school or equivalent  Some college, no degree  Associate degree  Bachelor's degree  Graduate degree (e.g. Master)  Doctoral or professional degree  Professional training  Other (specify) |
| **Income**  Select one  Less than $20,000  $20,000 to $34,999  $35,000 to $49,999  $50,000 to $74,999  $75,000 to $99,999  Over $100,000 |
| **Marital status**  Select one  Never married  Married  Divorced/widowed |
| **Do you have children?**  Select one  Yes  No |
| **I have good computer skills**  Select one  Strongly agree  Agree  Not sure  Disagree  Strongly disagree |
| **I prefer to wait a while before trying the latest medical technologies**  Select one  Strongly agree  Agree  Not sure  Disagree  Strongly disagree |
| **I believe in an afterlife**  Select one  Strongly agree  Agree  Not sure  Disagree  Strongly disagree |
| **Has someone close to you died recently?**  Select one  Yes  No |
| **My attitude towards death is**  Select all that apply  Death (not the process of dying) frightens me  I wish I could see what the future will be like  If I could remain youthful for another 100 years I would  If I could live indefinitely, even "forever", I would  Death will always be inevitable and science will never be able to change that  Death is natural and should not be avoided  None of the above  Other (specify) |
| **Have you heard of the practice of cryopreservation before?**  Select one  Yes, more than most people  Yes, about as much as most people  No  Unsure |
| **Have you decided what you want to happen to your body when you die?**  Select one  No, and I don't plan to soon  No, but I will soon  Yes, burial  Yes, cremation  Yes, cryopreservation  Other (specify) |
| **What are the most important considerations in your death care arrangements?**  Select all that apply  Cost  Tradition  Convenience  Customer service  Family's wishes  Quality  Other (specify) |
| **Have you formally arranged for your death care / after-life wishes to be carried out?**  Select all that apply  No  Yes, I've told my family  Yes, I've made a will  Yes, I've selected a funeral home or cryopreservation provider  Yes, I've prepaid for services (e.g. funeral home or cryopreservation provider)  Other (specify)  *Visibility Logic*  Visible only when “Have you decided what you want to happen to your body when you die?” isn’t “No, but I will soon” and isn’t “no, and I don’t plan to soon” |
| **How have you learned about cryopreservation?**  Select all that apply  TV/Film (fiction)  TV/Film (documentary, news)  Word of mouth  Internet articles  Youtube  Cryopreservation provider website  Other (specify)  *Visibility Logic*  Visible only when “Have you heard of the practice of cryopreservation” isn’t “No” |
| **Have you ever actively sought out information about cryopreservation?**  Select one  Yes  No  *Visibility Logic*  Visible only when “Have you heard of the practice of cryopreservation” isn’t “No” |
| **Are you signed up for cryopreservation?**  Select one  Yes, long time ago  Yes, recently  No - but currently signing up  No - but interested  No - never thought about it  No - not interested  None of the above  *Visibility Logic*  Visible only when “Have you heard of the practice of cryopreservation” isn’t “No” |
| **If anything, what would convince you to sign up for cryopreservation?**  Text answer |
| **How many cryopreservation companies provide service in your country?**  Numerical response |
| **Why is 'vitrification' employed in most cases?**  Select one  To increase oxygen uptake  during cool down  To enlarge blood vessels  To cool down faster  To reduce ice formation  I don't know |
| **How many people have been cryopreserved?**  Numerical answer |
| **How much would you guess it costs (USD) to have your whole body cryopreserved?**  Numerical answer |
| **In how many years do you think it will be possible to read most memories from a cryopreserved brain, in an ideal case?**  Numerical answer |
| **In how many years do you think it will be possible to revive a cryopreserved body, in an ideal case?**  Numerical answer |
| **Why have you not signed up yet / earlier?**  Select all that apply  Too expensive (monthly cost)  I live in a country far away from all providers  Too complex / I don't know how  Social stigmata  I don't trust the provider(s)  Takes too much time  Can do it later  Never thought about it until now  Other (specify)  *Visibility logic*  Visible only if “Are you signed up for cryopreservation” is “No - but interested”, “No - but currently signing up”, “Yes, recently” |
| **Are you a member of a cryopreservation group/community?**  Select one  Yes  No |
| **Are you a member of a longevity or life extension group/community?**  Select one  Yes  No |
| **Choose statements you agree with**  Select all that apply  Cryopreservation is exciting  There's a good chance cryopreservation will work  I can afford cryopreservation  coverage  Cryopreservation is immoral  Some cryopreservation  companies are trustworthy  Cryopreservation will create  overpopulation  You can't trust most  cryopreservation companies  Cryopreservation makes death  harder for my loved ones  Cryopreservation is unlikely to  work  None of the above  Other (specify) |
| **Cryopreservation should be illegal**  Select one  Strongly agree  Agree  Not sure  Disagree  Strongly disagree |
| **What are the biggest problems with cryopreservation today?**  Select all that apply  Cryopreservation damages the  body in a relevant manner / too much  The cryopreservation company  might go out of business and the bodies will thaw  No one in the future will want to  revive the bodies  Very few scientists and doctors  recommend the practice  It is too expensive  It carries a social stigma  None / Nothing relevant |
| **Most people feel that cryopreservation is…**  Select all that apply  Cool and exciting, if quite  unusual  A scam/rip-off  Promising technology  Selfish  Should be illegal  Good chance it works  Just not interesting  Weird  Dystopian  None of the above |
| **Select your biggest concerns about cryopreservation**  Select all that apply  I don't want to be woken up in a  dystopian future  I will not understand the society  I wake up in  My friends and family would not  be with me when I wake up  I might be poor after I wake up  It will hurt or be traumatic  The chance that it will work is  low  I don't have any relevant  concerns  Other (specify)  *Visibility Logic*  Visible only when “Are you signed up for cryopreservation” isn’t “No - not interested” |
| **I would be significantly more likely to sign up for cryopreservation if**  Select all that apply  None, I'm already very  interested  It costs the same as cremation  My loved ones sign up too  My physician recommended it  A major healthcare provider  offered cryonics  I could specify under what  conditions to revive me or not  I could opt to donate my  memories instead of being  revived  None, not interested at all  Other (specify) |
| **What do you think are the most significant risks to cryopreserved bodies being revived?**  Select all that apply  Company goes out of business  Future generations forget about  you  Cryopreservation is made illegal  Revival is too expensive  Current cryopreservation is too  crude  Future society does not develop  technologically  It is impossible in principle  None of the above |
| **At what monthly price (USD) is getting cryopreservation coverage a no-brainer?**  Numerical answer  *Visibility Logic*  Visible only when “Are you signed up for cryopreservation” isn’t “No - not interested” |
| **Which is most important to you when choosing a cryopreservation provider?**  Select all that apply  Cost  Case response capabilities  Research  Track record (age, # of patients)  Ease of signing up  Customer service  Corporate governance  Financial stability  Aldehyde-stabilized cryopreservation  (Not interested under any  circumstances)  Other (specify)  *Visibility Logic*  Visible if “Are you signed up for cryopreservation” isn’t “No - not interested” |
| **If / once possible, in what form would you like to be revived?**  Select all that apply  Biological (flesh-based) body  Whatever is standard at the  time  Depends on factors such as  quality of experience,  maintenance cost,  convertibility between forms  Wake me up mid-process and  ask (provide information about  current norms)  Digital avatar (corporeal)  Digital upload (not necessarily  corporeal)  Whatever other persons  (revived or still living) who  know me recommend  Whatever becomes available  first  Whatever is less costly  (Not interested under any  circumstances)  *Visibility Logic*  Visible only when “Are you signed up for cryopreservation” isn’t “No - not interested” |
| **I believe that cryopreservation is an exciting idea and intend on looking into it further / or I'm already sign up**  Multiple choice  Strongly agree  Agree  Not sure  Disagree  Strongly disagree |
| **I would be interested in talking to a cryopreservation company to discuss signing up**  Multiple choice  Strongly agree  Agree  Not sure  Disagree  Strongly disagree |
